# Enhancement of fermentable sugar yields by α-xylosidase supplementation of commercial cellulases

**DOI:** 10.1186/1754-6834-6-58

**Published:** 2013-04-26

**Authors:** Dina Jabbour, Melissa S Borrusch, Goutami Banerjee, Jonathan D Walton

**Affiliations:** 1 Department of Energy Great Lakes Bioenergy Research Center, Michigan State University, East Lansing, MI, 48824, USA; 2 Department of Energy Plant Research Laboratory, Michigan State University, East Lansing, MI, 48824, USA; 3 Current address: Codexis, Inc., 200 Penobscot Dr., Redwood City, CA, 94063, USA

**Keywords:** *Aspergillus niger*, *Trichoderma reesei*, Biofuel, Lignocellulose, Xyloglucan, Cellulase, Corn stover

## Abstract

**Background:**

Although α-linked xylose is a major constituent of the hemicelluloses of land plants, few secreted α-xylosidases have been described from fungi or bacteria. AxlA of *Aspergillus niger* is a secreted α-xylosidase that was earlier shown to promote the release of free glucose (Glc) and xylose (Xyl) from substrates containing α-linked xylose, including isoprimeverose (IP), the heptasaccharide subunit of pea xyloglucan (XG), and tamarind XG.

**Results:**

The utility of AxlA for enhancing release of free Glc and Xyl in combination with commercial enzyme cocktails from dicotyledonous and monocotyledonous plants was examined. Without AxlA supplementation, a mixture of CTec2 and HTec2 (both of which are derived from *T. reesei*) did not release significant levels of Glc from pea XG or tamarind XG. This is consistent with their lack of detectable α-xylosidase activity using model substrates. On alkaline hydrogen peroxide-pretreated corn stover, supplementation of CTec2/HTec2 (at a loading of 2.5 mg/g glucan) with AxlA (at a loading of 8 mg/g glucan) increased Glc yields from 82% to 88% of the total available Glc and increased Xyl yields from 55% to 60%. AxlA supplementation also improved Glc yields from corn stover treated with the commercial cellulase Accellerase 1000. The AxlA enhancement was not a general protein effect because bovine serum albumin or bovine gamma-globulin at similar concentrations did not enhance Glc yields from corn stover in response to CTec2/HTec2. Supplementation of CTec2/HTec2 with AxlA did not enhance Glc release from pretreated green or etiolated pea tissue. However, AxlA did enhance Glc and Xyl yields compared to CTec2/HTec2 alone from another dicotyledonous herbaceous plant, *Chenopodium album* (lamb’s quarters).

**Conclusion:**

Supplementation of commercial cellulase cocktails with AxlA enhances yields of Glc and Xyl from some biomass substrates under some conditions, and may prove useful in industrial lignocellulose conversion.

## Background

The fermentative production of biofuels from lignocellulosic materials requires the efficient deconstruction of plant polysaccharides to free sugars. This process can be catalyzed by complex mixtures of enzymes containing cellulases, xylanases, and other glycosidases. Although enzymatic depolymerization is specific, nondestructive, and environmentally benign, enzyme cost is still a major barrier to the development of an economical lignocellulosic biofuels industry. Enzyme mixtures could theoretically be improved by a number of means, such as optimizing the relative proportions of the necessary enzyme activities, increasing specific activity of the individual enzymes, or improving their stability. Another way in which enzyme mixtures could be improved is to supplement them with accessory enzymes that contribute novel catalytic activities or other functions [1].

In addition to cellulose, all plant cell walls contain a heterogenous assortment of complex polysaccharides known as hemicelluloses. Xyloglucans (XG), which are present in the primary walls of terrestrial plants, comprise a β1,4-linked glucan backbone substituted with α-linked xylose, and in some plants additionally with galactose (Gal) and fucose (Fuc) [2-4]. XG is the major hemicellulose in the primary walls of dicotyledenous plants but is also present in cereals [5]. Commercial “cellulase” preparations, mostly derived from *Trichoderma reesei*, lack α-xylosidase activity, and the genome of this fungus is not predicted to contain any genes encoding secreted α-xylosidases [6]. In contrast, the soft rot fungus *Aspergillus niger* secretes an α-xylosidase, AxlA, which is active against *p*-nitrophenyl-α-xyloside (pNPαX), isoprimeverose (IP), and an XG-derived heptasaccharide isolated from pea walls [6]. In combination with xyloglucanase, β-glucosidase, and β-galactosidase, AxlA efficiently released free Glc from tamarind XG [6]. Because the action of AxlA releases both Glc and Xyl from XG, inclusion of AxlA might have utility in improving the efficiency of commercial enzyme mixtures on realistic biomass substrates, including corn stover.

## Results

### Commercial cellulases do not degrade xyloglucan because they lack α-xylosidase

**Figure 1:**
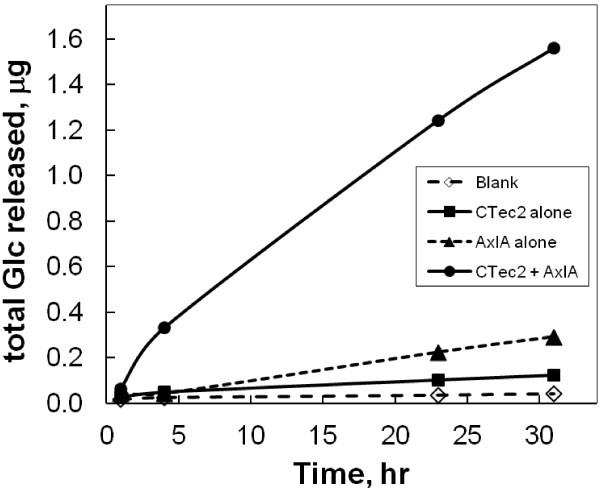
**AxlA supplementation of CTec2/HTec2 enhances release of free Glc from pea xyloglucan (XG).** Each reaction contained 10 μg XG, 50 ng of a 75:25 mixture of CTec2/HTec2, and 80 ng AxlA in a total volume of 50 μl. The free Glc content in 10 μl of the reaction mixture was measured at each time point by an enzyme-linked assay.

**Figure 2:**
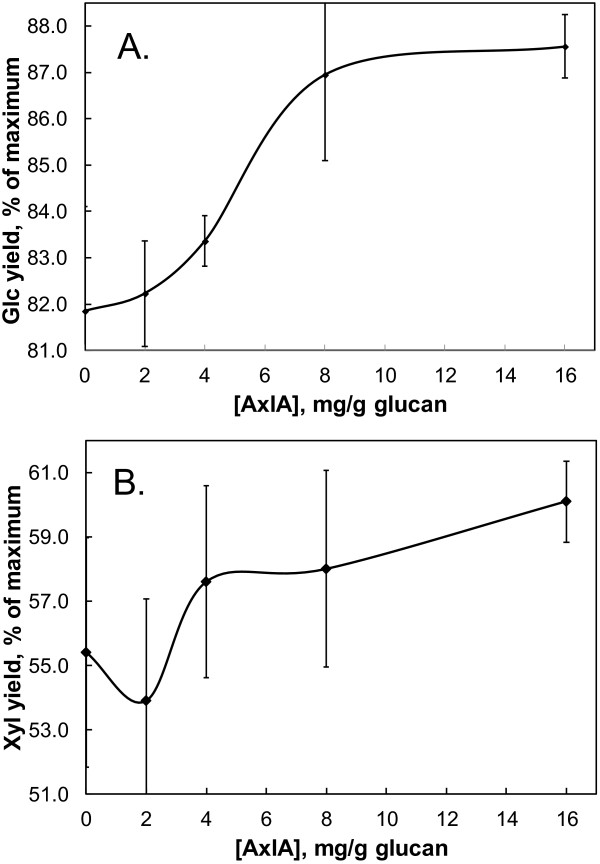
**Glc release from tamarind XG as a function of AxlA concentration.** The 75:25 CTec2/HTec2 loading was 2.5 mg/g glucan and the reaction volume was 500 μl. The XG concentration was 3 mg/ml, giving a maximum possible yield of 1.5 mg Glc/ml.

In mixtures of pure enzymes (i.e., β-glucosidase, β-galactosidase, and xyloglucanase), AxlA was required for release of free Glc and Xyl from isolated pea XG fragments and from tamarind XG [6]. Likewise, supplementation with AxlA was required for the release of free Glc from intact pea XG in response to the commercial cellulase cocktails CTec2 and HTec2 (Figure 1). Addition of AxlA enhanced Glc yield by 18-fold in 30 hr compared to CTec2/HTec2 alone. These results are consistent with the earlier results showing the absence of α-xylosidase activity in CTec2 or HTec2 against the model substrate pNPαX and the disaccharide IP [6]. These results furthermore indicate that a combination of CTec2 and HTec2 has all of the necessary enzymes to degrade pea XG except α-xylosidase. In this regard they are similar to the commercial product Driselase from the basidiomycetous fungus *Irpex lacteus*, which degrades XG only to IP [7,8]. AxlA supplementation was also necessary for complete depolymerization of tamarind XG by CTec2/HTec2 (Figure 2). Tamarind XG is less fucosylated but more galactosylated than pea XG, but both contain α-linked xylose. In the absence of AxlA, CTec2/HTec2 released almost no Glc even in 48 hr. An AxlA to CTec2/HTec2 ratio of 1 to 3 (on a protein mass basis) was near saturating for release of Glc in 48 hr (Figure 2).

**Figure 3:**
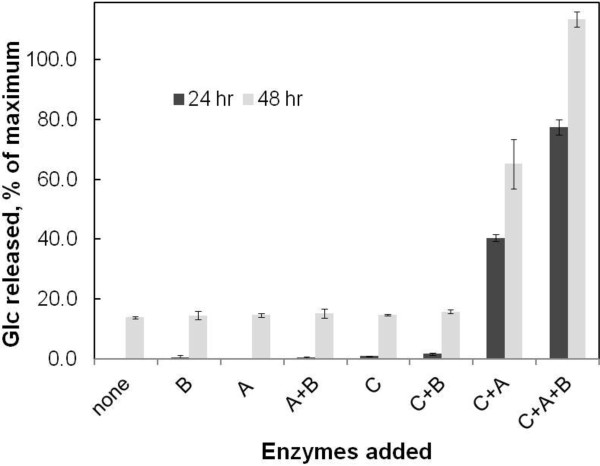
**Supplementation with β-galactosidase further improves Glc yields.** The tamarind XG concentration was 3 mg/ml, the CTec2/HTec2 (75:25) loading was 2.5 mg/g glucan, and the AxlA and β-galactosidase loadings were each 8 mg/g glucan. A, AxlA; B, β-galactosidase; C, CTec2/HTec2.

Although addition of AxlA to CTec2/HTec2 greatly stimulated release of free Glc, yields of Glc were still only about half of the maximal possible (Figure 2). A possible explanation for this is that β-galactosidase activity was limiting in these reactions, and therefore any Glc moiety substituted with Gal as well as Xyl would not be released. In fact, addition of both β-galactosidase and AxlA to CTec2/HTec2 stimulated Glc release compared to reactions without β-galactosidase (Figure 3). This experiment indicates that CTec2/HTec2 is sub-optimal in regard to β-galactosidase as well as α-xylosidase for the digestion of tamarind XG.

### AxlA supplementation improves Glc yields from real biomass substrates

**Figure 4:**
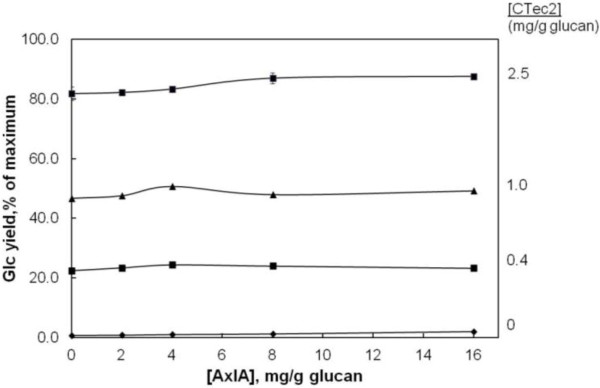
**AxlA supplementation improves Glc yields from pretreated corn stover by CTec2.** In this experiment, only CTec2 was used; similar results were obtained with a 75:25 ratio of CTec2/HTec2. The CTec2 loading was 0, 0.4, 1.0, or 2.5 mg/g glucan and the incubation time was 48 hr.

**Figure 5:**
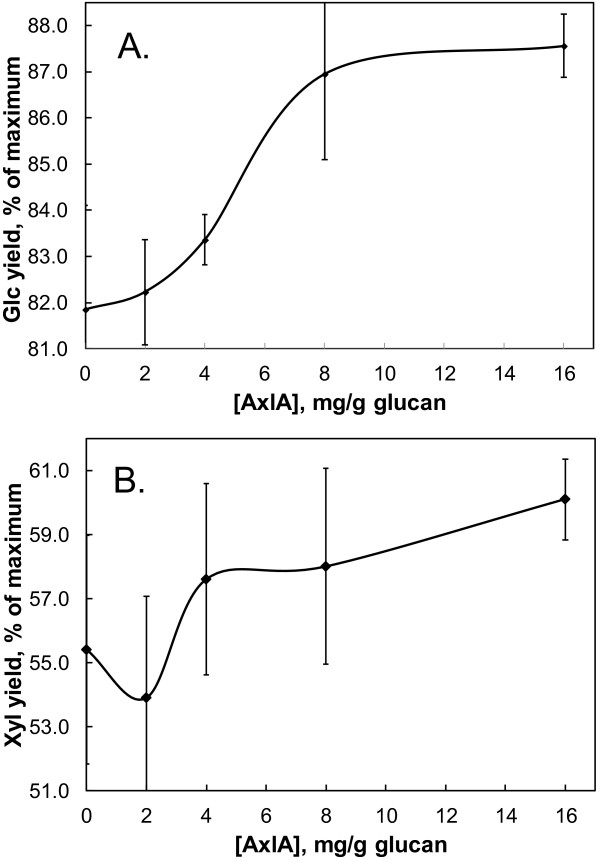
**Effect of AxlA supplementation on Glc and Xyl yields, shown in expanded scale.** (**A**) Results from the experiment shown in Figure 4 for 2.5 mg CTec2/g glucan. (**B**) Xyl release from the same experiment shown in expanded scale.

The effect of AxlA supplementation of CTec2/HTec2 on digestion of a biofuels-relevant biomass substrate, alkaline hydrogen peroxide (AHP)-pretreated corn stover, is shown in Figure 4. Because cellulose is the major form of Glc in corn stover and CTec2/HTec2 has strong cellulase activity, as expected Glc yields even without AxlA supplementation were high (Figure 4). At lower CTec/HTec2 loadings (i.e., 0.4 and 1.0 mg/g glucan), there was no apparent enhancement of Glc release by addition of AxlA (Figure 4). At the highest CTec2/HTec2 loading tested (2.5 mg/g glucan), however, there was a statistically significant increase in Glc yield after hydrolysis for 24 hr (data not shown) and 48 hr (Figure 4). Figure 5A shows the results from Figure 4 in expanded scale to highlight the enhancement by AxlA. Glc yields increased from 81.9 ± 1.5% to 87.5 ± 1.1% of the maximum possible Glc content at AxlA loadings above 8 mg/g glucan. Xyl yields were also increased by AxlA supplementation, as shown in expanded scale in Figure 5B. The AxlA effect on Xyl yield (5.1 ± 1.5% ) was statistically significant only at the highest CTec2/HTec2 (2.5 mg/g glucan) and AxlA loadings (16 mg/g glucan) tested.

**Figure 6:**
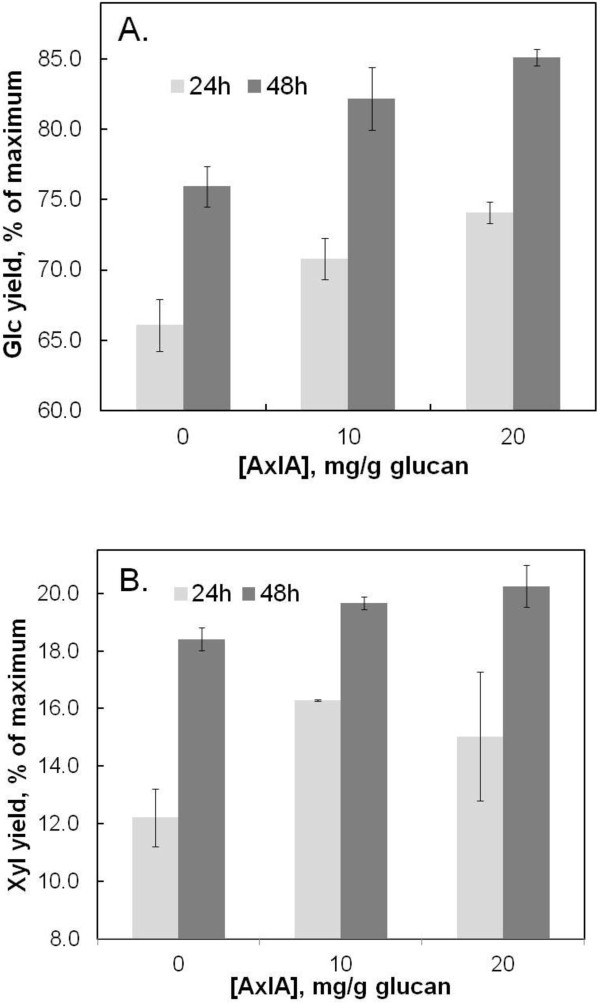
**AxlA supplementation enhances activity of Accellerase 1000.** (**A**) Glc yield at 24 hr and 48 hr hydrolysis times from AHP-pretreated corn stover in response to 5 mg/g glucan Accellerase 1000 and the indicated concentrations of AxlA. (**B**) Xyl yields from the same experiment.

AxlA also enhanced yields of Glc and Xyl from pretreated corn stover in response to another commercial cellulase, Accellerase 1000 (Figure 6). In 48 hr, AxlA increased Glc yields by 9.0 ± 1.2% (from 76 ± 2% to 85 ± 0.5% of maximum possible yield) and Xyl yields by 1.8 ± 1.1% (Figure 6).

### Time course of Glc release

**Figure 7:**
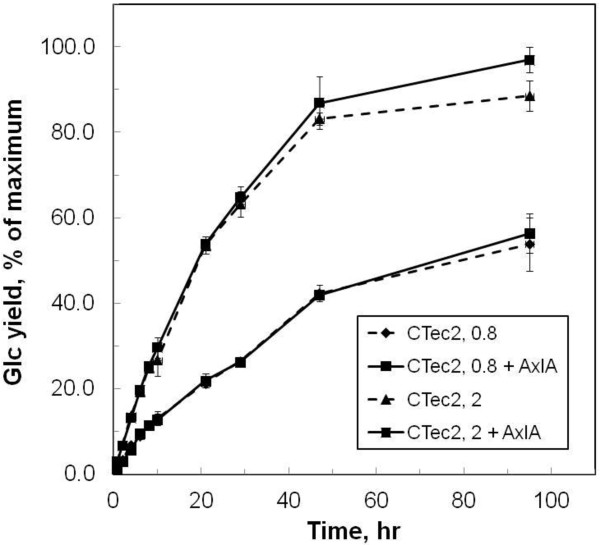
**Time course of enzymatic hydrolysis of pretreated corn stover with or without AxlA supplementation.** The CTec2/HTec2 (75:25) loading was 0.8 or 2 mg/g glucan and the AxlA loading was 4 mg/g glucan.

The release of Glc was monitored over 95 hr at two CTec2/HTec2 (75:25) loadings with and without AxlA. As expected, the higher CTec2/HTec2 loading released more Glc more quickly (Figure 7). At the lower CTec2/HTec2 loading, AxlA caused a small enhancement of Glc yield only at the highest AxlA loading, and this was not statistically significant (Figure 7). Likewise at the higher CTec2/HTec2 loading, a stimulatory effect of AxlA was seen only at 95 hr, although in this case the effect was statistically significant. Under these conditions, AxlA supplementation resulted in an 8.3% absolute increase in Glc yield, from 84 ± 1.9% to 92.3 ± 1.1% (Figure 7).

### Enhancement by AxlA is not a general protein effect

**Figure 8:**
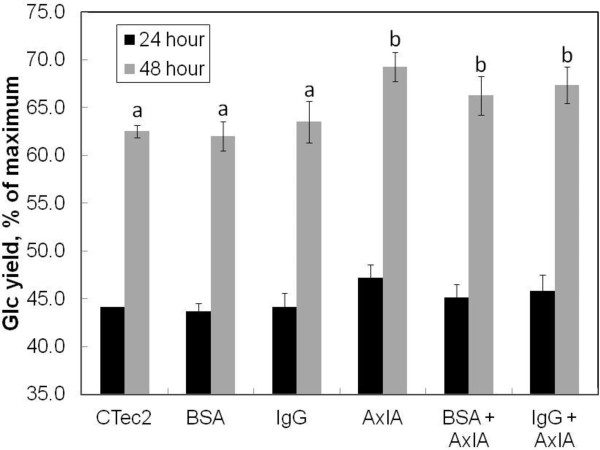
**Enhancement by AxlA is not a general protein effect.** All experiments used a 75:25 mixture of CTec2/HTec2 at a loading of 2.5 mg/g glucan in addition to the indicated component. “CTec2” indicates CTec/HTec2 alone. The loadings of AxlA, bovine serum albumin (BSA), and bovine immunoglobulin (IgG) were each 8 mg/g glucan. Lowercase letters above the data bars indicate significantly different or not from CTec2/HTec2 alone (P<0.05 in Tukey’s multiple comparison test, n=6).

Nonenzymatic proteins, such as bovine serum albumin (BSA), enhance apparent hydrolysis activity, probably by reducing nonspecific and/or nonproductive binding of cellulases and other enzymes to lignin [9]. To test whether the enhancement by AxlA might be due to a nonspecific protective effect on cellulases as opposed to its intrinsic enzymatic activity, we compared the effect on hydrolysis enhancement of AxlA against BSA and bovine gamma-globulin. As shown in Figure 8, neither BSA nor IgG stimulated Glc yields in response to CTec2/HTec2, nor did either protein affect the enhancement by AxlA (Figure 8). Furthermore, AxlA that had been boiled to destroy its activity did not stimulate Glc or Xyl release from corn stover (data not shown). Therefore, we conclude that its AxlA enhancement is due to the α-xylosidase activity and is not a general nonspecific protein effect.

### Response of herbaceous dicotyledons to AxlA supplementation

Corn stover, like other plants in the Poaceae, are generally considered to have lower levels of XG than dicotyledons and non-graminaceous monocotyledons [5]. To test whether herbaceous dicotyledons might therefore respond differently to AxlA supplementation, we tested dark-grown (etiolated) peas, light-grown (green) peas, and wild lamb’s quarters. Peas were chosen because their primary wall XG has been well-characterized [10]. Lamb’s quarters was chosen because, as a soft annual, it should have a high primary wall content. This is consistent with its Glc/Xyl ratio of ~5.7, which is close to etiolated and green peas (ratios of 5.4 and 5.6, respectively) and higher than corn stover (ratio 2.0) (Table 1).

**Table 1:** Monomer sugar composition of plant materials used in this study

**Plant material**	**Glc**	**Xyl**	**Ara**^**a**^	**Man**^**a**^	**Gal**	**Total**
**pea XG**	226 ± 19.0	287 ± 25.2	127 ± 8.1	8.5 ± 0.6	75 ± 6.0	72.4%
**tamarind XG**^**b**^	471 ± 8.3	351 ± 9.2	23 ± 2.1	0.0	155 ± 5.3	100%
**etiolated peas**	281.6 ± 12.0	49.4 ± 4.4	55.8 ± 4.8		48.3 ± 2.2	43.5%
**green peas**	106.7 ± 12.0	19.6 ± 0.7	30.8 ± 2.0		23.7 ± 2.7	18.1%
**corn stover**	391.5 ± 0.35	194.7 ± 10.9	33.3 ± 5.3		9.4 ± 2.3	62.9%
**lamb’s quarters**	170.1 ± 1.7	30.2 ± 0.03	24.6 ± 0.64		14.4 ± 0.14	23.9%

^a^The HPLC protocol could not resolve Ara and Man. Pea and tamarind XG’s were analyzed by gas chromatography of alditol acetates after hydrolysis with trifluoroacetic acid [17].

^b^From ref. 6.

All values (± 1 SD, n = 3) are expressed as mg/g dry weight. Materials were dried but otherwise not processed by washing or other fractionation before acid hydrolysis. “Total” indicates the percentage of the original dry weight accounted for by the indicated neutral sugars.

**Figure 9:**
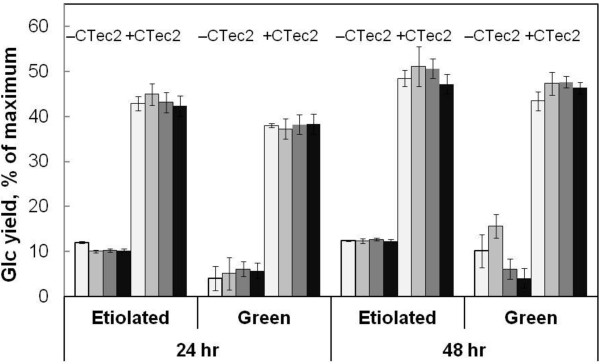
**AxlA supplementation does not enhance Glc yield from pea biomass.** Etiolated (dark-grown) and green (light-grown) peas (all of the above-ground parts) were not washed before or after AHP pretreatment. “+CTec2” indicates 15 mg/g glucan 75:25 CTec2/HTec2; “-CTec2” indicates neither CTec2 nor HTec2. Within each group of four data bars, the AxlA loadings from left to right were 0, 4, 8, and 16 mg/g glucan.

**Figure 10:**
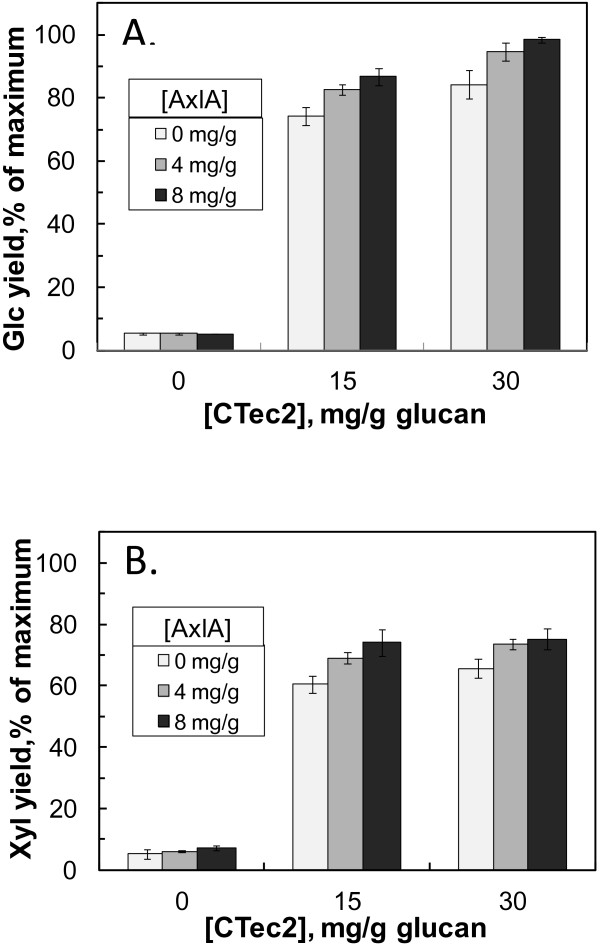
**AxlA supplementation enhances Glc and Xyl yields from lamb’s quarters.** (**A**) Glc yield, (**B**) Xyl yield. The CTec2/HTec2 ratio was 75:25. Hydrolysis time was 48 hr.

Yields of Glc from etiolated and light-grown pea were low, never exceeding 50% of available Glc content (Figure 9). Under no conditions tested did AxlA increase Glc yields from either kind of pea (Figure 9). However, AxlA supplementation did have a strong positive effect on Glc yields from lamb’s quarters, although higher loadings of CTec2/HTec2 were necessary to obtain Glc yields comparable to corn stover (Figure 10). At 30 mg/g CTec2/HTec2, a loading of 4 mg/g AxlA enhanced Glc yields from 82.2 ± 2.1% to 96.5 ± 0.4% (an absolute increase of 14.3%) and Xyl yields by 65.9 ± 0.8% to 75.5 ± 0.9% (an absolute increase of 9.6%) (Figure 10). Therefore, AxlA supplementation enhanced Glc and Xyl yields from some but not all herbaceous dicotyledons.

### Inhibition of AxlA by the products of enzyme hydrolysis

In general, a high level of AxlA was needed to see enhancement of Glc and Xyl release (Figures 5, 6). One possible explanation for this is that AxlA is inhibited by free Glc or Xyl or by compounds released from corn stover. Inhibition by Glc and Xyl was tested using pNPαX as substrate. The observed K_i_ for Glc was 109 mM (95% confidence interval: 85.4 – 132.3 mM) and the K_i_ for Xyl was 13.7 mM (confidence interval: 10.7 – 16.7 mM). These results predict that AxlA would be inhibited < 8% by the highest Xyl concentration achieved in our experiments. At higher biomass loadings, which would result in higher concentrations of these two sugars, inhibition of AxlA might become significant, as it would also for other hydrolytic enzymes such as cellobiohydrolase and endo-glucanase.

The possible inhibition by other compounds in the hydrolysate was also tested. Lamb’s quarters was digested with CTec2/HTec2 and the crude hydrolysate tested for inhibition of AxlA activity on pNPαX. At a hydrolysate loading of 30% (v/v), pNPαX hydrolysis by AxlA was inhibited less than 5%. We therefore conclude that inhibition by Glc, Xyl, or other compounds present in the hydrolysate does not account for the requirement for a relatively high concentration of AxlA.

## Discussion

All plant cell walls contain α-linked xylose, and commercial cellulase preparations derived from *T. reesei* lack α-xylosidase activity [6]. Like other micro-organisms (e.g., *Lactobacillus pentosus*; ref. [11]), in nature *T. reesei* probably deconstructs XG to the disaccharide isoprimeverose (IP), imports IP with a disaccharide transporter, and then converts it to free Xyl and Glc utilizing an intracellular α-xylosidase. This scenario, although unproven, is consistent with the large number of predicted enzymes of CAZy family GH31 lacking signal peptides found throughout the Ascomycota and Basidiomycota [6]. Because wild type brewer’s yeast cannot metabolize IP or other disaccharides, the lack of α-xylosidase in commercial cellulase preparations results in the loss of the energy content of the Glc and Xyl that remains locked up in IP [6].

The purpose of the current work was to test the hypothesis that supplementation of commercial cellulase mixtures with the secreted α-xylosidase of *A. niger* (known as AxlA) would improve Glc and Xyl yields under otherwise identical hydrolysis conditions. This would translate into higher ethanol yields from a given mass of lignocellulosic material, especially since the action of AxlA produces both Xyl and Glc. Consistent with this hypothesis, we found that supplementation of two commercial cellulase cocktails with AxlA resulted in higher yields of Glc and Xyl from corn stover and lamb’s quarters. Our results also indicate that under some conditions β-galactosidase activity in current commercial cellulases is limiting.

A noticeable trend in our experiments was that the stimulatory effect of AxlA supplementation was apparent only, or more so, when Glc yields were high as a result of higher CTec2/HTec2 loadings (Figures 4, 7, 10), longer hydrolysis times (Figure 7), or lower biomass recalcitrance (Figure 10). There are several possible explanations for this observation. The most probable explanation is that AxlA is a late-acting “terminal” enzyme that depends on other XG-active enzymes (e.g., xyloglucanase and β-galactosidase) to provide its substrate. If those enzymes do not act efficiently, then AxlA has no substrate on which to act itself. Another possible explanation is that access to XG is occluded by other wall polymers, perhaps including cellulose. In a recently proposed model of the structure of the primary wall, much of the XG is hypothesized to be appressed between or embedded within cellulose microfibrils rather than spanning cellulose microfibrils as in the original “tethered network” model [12]. If so, then the enzymes active on XG could not gain access to their substrate(s) until after the cellulases (i.e., cellobiohydrolases and endo-β1,4-glucanases) had a chance to act. Hence, AxlA would have an effect on Glc and Xyl yields only at the terminal stages of wall deconstruction. This could also explain the lack of an effect of AxlA supplementation on Glc yields from pea cell walls, postulating that insufficient hydrolysis of cellulose by cellulases (manifested by relatively low yields of Glc) blocks access by the XG-active enzymes, including AxlA, to the XG.

Another general observation from these studies was that a high level of AxlA protein had to be added relative to the levels of commercial enzymes in order to see an enhancement. The ratio of AxlA to commercial enzyme (on a protein mass basis) varied from 0.3 to 6.4 in different experiments. There could be several explanations for this. It does not seem to be due to intrinsically low activity, because the K_m_ and k_cat_ of AxlA on model substrates were comparable to other glycoside hydrolases [6]. Experiments reported herein exclude inhibition by Glc, Xyl, or other hydrolysate components as an explanation. The need for high loadings of AxlA could be improved by bioprospecting or protein engineering to identify or create an α-xylosidase with higher specific activity. Another possible approach to reduce α-xylosidase loading would be to identify an enzyme with a pH optimum closer to those of the *T. reesei* enzymes, i.e, pH 4.6 - 4.8, rather than its current pH optimum of ~3.7 [6].

Even though cereals are widely believed to contain smaller amounts of XG compared to dicotyledonous plants, AxlA supplementation was as effective on corn stover as it was on lamb’s quarters, and more effective than on peas. The explanation for this might lie in our imperfect knowledge of the XG content of cereals. Because of its lower degree of sidechain substitution (by Xyl, Gal, and/or Fuc), cereal XG is less water-soluble, harder to extract, and more difficult to distinguish by linkage analysis from pure β1,4-glucans such as cellulose. It has been proposed that the levels of XG in cereals may, in fact, be higher than commonly believed, and perhaps even comparable to the levels found in dicotyledons [2,3,13].

## Conclusions

Secreted, fungal α-xylosidase (AxlA) is a recently described cell wall degrading enzyme that is necessary for the release of free Glc and Xyl from XG. Supplementation of commercial cellulase preparations with AxlA may, at least in some circumstances, significantly impact the overall efficiency of biofuels production from lignocellulosic materials.

## Methods

### Plant materials and pretreatments

Stover of corn (*Zea mays* L.) was ground to 0.5 mm particle size with a Wiley mill before pretreating with alkaline hydrogen peroxide (AHP) as described [14]. AHP conditions were 10% biomass loading, 0.5 g H_2_O_2_/g biomass, and shaking at 90 rpm and 24°C for 24 hr. Peas (*Pisum sativum* L. “Little Marvel”) were soaked in water for 24 hr with bubbling air and grown in vermiculite either in either total darkness for 5-7 days (“etiolated peas”) or in a greenhouse for 9-14 days (“green peas”). After freeze-drying, the etiolated plants were ground in liquid nitrogen. The green peas were lyophilized and then ground in a Wiley mill to pass a 0.5-mm screen. Both were then pretreated by the same AHP conditions used for corn stover. *Chenopodium album* L. (lamb’s quarters) was collected from local abandoned fields in mid-August. Plants were dried at 50°C and ground in a Wiley mill to pass a 0.5 mm screen and pretreated by AHP.

Pea XG was prepared as described [15,16] and its composition was analyzed by the alditol acetate method [17]. It was judged to be partially pure by its atypical content of Ara and because the sum of the neutral sugars did not add up to 100% (Table 1). Tamarind XG was purchased from Megazyme, Inc. (Wicklow, Ireland). Its composition was close to the manufacturer’s specifications [6].

### Cell wall analysis

Cell wall sugar composition (of materials other than pea XG) was determined by two-stage hydrolysis with sulfuric acid without prior removal of extractives [18]. Sugars were separated by HPLC using a Bio-Rad (Hercules, CA) Aminex HPX-87P column at 80°C with 1 ml/min water as mobile phase and detection by refractive index. Each run took about 20 min. Under these conditions, Ara and Man could not be resolved and are reported together. Because the biomass was not washed to remove extractives prior to acid hydrolysis, the compositional analysis includes any contributions from starch, sucrose, free monomeric sugars, or acid-labile conjugated Glc and Xyl. Recovery from the acid hydrolysis step was calculated to be 95% for Glc, Ara, and Gal, and 85% for Xyl.

### Enzymes

Cellic CTec2 (lot number VCPI0004) and HTec2 (lot number VHN00002) were obtained from Novozymes, Inc. (Davis, CA) and typically used at a ratio of 3:1 on a protein mass basis. The protein concentrations of CTec2 and HTec2 were determined to be 130 mg/ml and 101 mg/ml, respectively, by the dye-binding assay of Bradford [19] using bovine IgG as standard. The CTec2/HTec2 enzyme mixture was typically freshly diluted 500-fold with 50 mM sodium citrate, pH 4.8, and used at a final protein concentration of 2.5 mg/g glucan. Accellerase 1000 (lot number 1600844643; 69 mg protein/ml) was obtained from Genencor, Inc. (now DuPont Industrial Biosciences, Palo Alto, CA) and diluted similarly. AxlA from *Aspergillus niger* (GenBank BK008484.1) was prepared by expression in *Pichia pastoris* as described and stored in aliquots at -80°C in 50 mM sodium acetate + 20% glycerol, pH 5 [6]. The other pure enzymes, all derived from *T. reesei*, were obtained commercially or prepared by expression in *P. pastoris* as described [20,21].

### Enzyme assays

Unless other specified, enzyme hydrolysis reactions were performed in 96-well deep-well plates in a reaction volume of 0.5 ml, as described [20]. Glucan concentration was typically 2 mg/ml. The buffer was 50 mM sodium citrate, pH 4.8, containing 25 μg/ml each of tetracycline and cycloheximide. Assays were run in duplicate, sampled twice, and the Glc and Xyl levels measured twice. Therefore, each data point represents the mean of eight values. All error bars represent +/- one standard deviation of the mean.

Glc and Xyl were measured using enzyme-linked colorimetric assays (Megazyme kits K-GLUC and K-XYLOSE, respectively). These assays detect only free Glc and Xyl and not cellobiose or oligomeric sugars.

For competition studies, pNPαX was tested at 0, 1, 2.5, 5, 10, 15, and 20 mM, and Glc and Xyl were tested at 0, 10, 25, 50, and 100 mM [6]. All reaction volumes were 100 μl. Inhibition constants (K_i_) were calculated using nonlinear curve fitting with GraphPad Prism (San Diego, CA). Hydrolysate for testing inhibition by hydrolysate was prepared by digesting lamb’s quarters or corn stover (2 mg glucan/ml) for 24 hr with CTec2/HTec2 at 15 mg/g glucan. After centrifugation, the hydrolysate was added up to a concentration of 30% (v/v) to a 100 μl reaction volume containing pNPαX and AxlA.

## Abbreviations

AHP: Alkaline hydrogen peroxide; Ara: Arabinose; BSA: Bovine serum albumin; Fuc: Fucose; Gal: Galactose; Glc: Glucose; IgG: Immunoglobulin; IP: Isoprimeverose; pNPαX: *p*-nitrophenyl-α-xyloside; Xyl: Xylose; XG: Xyloglucan

## Competing interests

The authors declare that they have no competing interests.

## Authors’ contributions

DJ, MSB, GB, and JDW designed and performed the experiments. JDW and DJ wrote the paper. All authors read and approved the final manuscript.
